# 2-(2,5-Dimeth­oxy­phen­yl)-*N*-[2-(4-hy­droxy­phen­yl)eth­yl]acetamide

**DOI:** 10.1107/S1600536812008975

**Published:** 2012-03-07

**Authors:** Hyeong Choi, Yong Suk Shim, Byung Hee Han, Sung Kwon Kang, Chang Keun Sung

**Affiliations:** a Department of Chemistry, Chungnam National University, Daejeon 305-764, Republic of Korea; b Department of Food Science and Technology, Chungnam National University, Daejeon 305-764, Republic of Korea

## Abstract

In the title compound, C_18_H_21_NO_4_, the dihedral angles between the acetamide group and the meth­oxy- and hy­droxy-substitured benzene rings are 80.81 (5) and 8.19 (12)°, respectively. The benzene rings are twisted with respect to each other, making a dihedral angle of 72.89 (5)°. In the crystal, N—H⋯O and O—H⋯O hydrogen bonds link the mol­ecules into a three-dimensional network.

## Related literature

For general background to tyrosinase, see: Kubo *et al.* (2000 ▶). For the development of tyrosinase inhibitors, see: Lemic-Stojcevic *et al.* (1995 ▶); Battaini *et al.* (2000 ▶); Cabanes *et al.* (1994 ▶); Thanigaimalai *et al.* (2010 ▶).
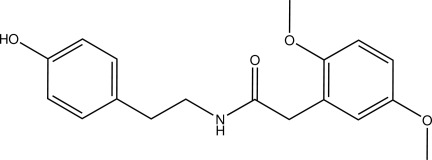


## Experimental

### 

#### Crystal data

C_18_H_21_NO_4_
*M* *_r_* = 315.36
Orthorhombic, 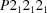

*a* = 8.1628 (8) Å
*b* = 12.0701 (11) Å
*c* = 17.0176 (16) Å
*V* = 1676.7 (3) Å^3^
*Z* = 4
Mo *K*α radiation
μ = 0.09 mm^−1^
*T* = 296 K
0.3 × 0.23 × 0.1 mm

#### Data collection

Bruker SMART CCD area-detector diffractometer
8417 measured reflections
3638 independent reflections
2352 reflections with *I* > 2σ(*I*)
*R*_int_ = 0.063

#### Refinement

*R*[*F* ^2^ > 2σ(*F* ^2^)] = 0.040
*wR*(*F* ^2^) = 0.088
*S* = 0.87
3638 reflections
216 parameters
H atoms treated by a mixture of independent and constrained refinement
Δρ_max_ = 0.11 e Å^−3^
Δρ_min_ = −0.12 e Å^−3^

### 

Data collection: *SMART* (Bruker, 2002 ▶); cell refinement: *SAINT* (Bruker, 2002 ▶); data reduction: *SAINT*; program(s) used to solve structure: *SHELXS97* (Sheldrick, 2008 ▶); program(s) used to refine structure: *SHELXL97* (Sheldrick, 2008 ▶); molecular graphics: *ORTEP-3 for Windows* (Farrugia, 1997 ▶); software used to prepare material for publication: *WinGX* (Farrugia, 1999 ▶).

## Supplementary Material

Crystal structure: contains datablock(s) global, I. DOI: 10.1107/S1600536812008975/tk5063sup1.cif

Structure factors: contains datablock(s) I. DOI: 10.1107/S1600536812008975/tk5063Isup2.hkl

Supplementary material file. DOI: 10.1107/S1600536812008975/tk5063Isup3.cml

Additional supplementary materials:  crystallographic information; 3D view; checkCIF report

## Figures and Tables

**Table 1:** Hydrogen-bond geometry (Å, °)

*D*—H⋯*A*	*D*—H	H⋯*A*	*D*⋯*A*	*D*—H⋯*A*
N10—H10⋯O19^i^	0.88 (2)	2.16 (2)	3.023 (2)	165.4 (19)
O19—H19⋯O9^ii^	0.87 (3)	1.76 (3)	2.6289 (19)	174 (3)

Symmetry codes: (i) 
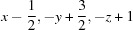
; (ii) 
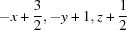
.

**Fig. 1.**
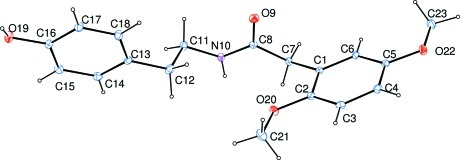
Molecular structure of the title compound, showing the atom-numbering scheme and 30% probability ellipsoids.

**Fig. 2.**
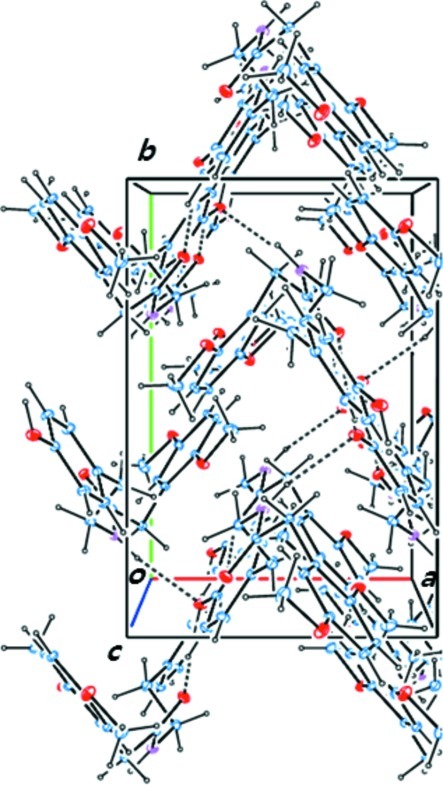
Part of the packing diagram of the title compound, showing a three-dimensional network of molecules linked by intermolecular N—H···O and O—H···O hydrogen bonds (dashed lines).

## supplementary crystallographic information

### Comment

Tyrosinase is a copper containing enzyme which acts as a catalyst in two different reactions involving the hydroxylation of monophenols to *o*-diphenols and the oxidation of the *o*-diphenols to *o*-quinones. This class of enzyme is widely distributed in the plant, animal and microorganism kingdoms (Kubo *et al.*, 2000), and its inhibition is one of the major strategies in developing new whitening agents. Over the last few decades, various tyrosinase inhibitors, including azelaic acid (Lemic-Stojcevic *et al.*, 1995), kojic acid (Battaini *et al.*, 2000), arbutin (Cabanes *et al.*, 1994), and *N*-phenylthiourea (PTU) (Thanigaimalai *et al.*, 2010) have been studied. But some of their individual activities are either not potent enough to be considered of practical use or not compatible with safety regulations for food and cosmetic additives. In our continuing search for tyrosinase inhibitors, we have synthesized the title compound, (I), from the reaction of 2,5-dimethoxyphenyl acetyl chloride and tyramine under ambient conditions. Herein, the crystal structure of (I) is described (Fig. 1).

The 2,4-dimethoxyphenyl and 3-hydroxyphenyl moieties are almost planar with r.m.s. deviations of 0.008 and 0.009 Å, respectively, from their corresponding least-squares planes. The dihedral angles between the acetamide group (C7–N10) and the benzene rings (C1–C6 + O20 and O22; and C12–O19) are 80.81 (5) and 8.19 (12)°, respectively. The benzene groups are twisted with respect to each other making a dihedral angle of 72.89 (5)°. The presence of intermolecular N10—H10···O19^i^ and O19—H19···O9^ii^ [symmetry codes: (i) *x* - 1/2, -*y* + 3/2, -*z* + 1, (ii) -*x* + 3/2, -*y* + 1, *z* + 1/2] hydrogen bonds link the molecules into a three-dimensional network (Fig. 2 and Table 1).

### Experimental

The starting materials, 2,5-dimethoxyphenyl acetyl chloride and tyramine, were purchased from Sigma Chemical Co. Solvents for organic synthesis were redistilled before use. All other chemicals and solvents were of analytical grade and were used without further purification. The title compound was prepared from the reaction of 2,5-dimethoxyphenyl acetyl chloride (0.21 g, 1.0 mmol) and tyramine (0.14 g, 1.0 mmol) by simple substitution in THF (6 ml) triethylamine (0.12 g, 1.2 mmol). The solvent was removed under reduced pressure. The mixture was purified by column chromatography on silica gel (2:1 dichloromethane/ethylacetate) to give the title compound. Colourless crystals were obtained by slow evaporation of its ethanol solution at room temperature.

### Refinement

H atoms of the NH and OH groups were located in a difference Fourier map and refined freely (N—H = 0.88 (2) Å and O—H = 0.87 (3) Å]. Other H atoms were positioned geometrically and refined using a riding model, with C—H = 0.93–0.97 Å, and with *U*_iso_(H) = 1.2*U*_eq_(C) for aromatic and methylene, and 1.5*U*_eq_(C) for methyl H atoms. In the absence of significant anomalous scattering effects, 1471 Friedel pairs were averaged in the final refinement.

### Crystal data

**Table:**

C_18_H_21_NO_4_	*F*(000) = 672
*M**_r_* = 315.36	*D*_x_ = 1.249 Mg m^−^^3^
Orthorhombic, *P*2_1_2_1_2_1_	Mo *K*α radiation, λ = 0.71073 Å
Hall symbol: P 2ac 2ab	Cell parameters from 2320 reflections
*a* = 8.1628 (8) Å	θ = 2.9–23.6°
*b* = 12.0701 (11) Å	µ = 0.09 mm^−^^1^
*c* = 17.0176 (16) Å	*T* = 296 K
*V* = 1676.7 (3) Å^3^	Block, colourless
*Z* = 4	0.3 × 0.23 × 0.1 mm

### Data collection

**Table:**

Bruker SMART CCD area-detector diffractometer	*R*_int_ = 0.063
Graphite monochromator	θ_max_ = 27.5°, θ_min_ = 2.8°
φ and ω scans	*h* = −4→10
8417 measured reflections	*k* = −15→7
3638 independent reflections	*l* = −18→21
2352 reflections with *I* > 2σ(*I*)	

### Refinement

**Table:**

Refinement on *F*^2^	Primary atom site location: structure-invariant direct methods
Least-squares matrix: full	Secondary atom site location: difference Fourier map
*R*[*F*^2^ > 2σ(*F*^2^)] = 0.040	Hydrogen site location: inferred from neighbouring sites
*wR*(*F*^2^) = 0.088	H atoms treated by a mixture of independent and constrained refinement
*S* = 0.87	*w* = 1/[σ^2^(*F*_o_^2^) + (0.0365*P*)^2^] where *P* = (*F*_o_^2^ + 2*F*_c_^2^)/3
3638 reflections	(Δ/σ)_max_ < 0.001
216 parameters	Δρ_max_ = 0.11 e Å^−^^3^
0 restraints	Δρ_min_ = −0.12 e Å^−^^3^

### Special details

**Table:**

Geometry. All s.u.'s (except the s.u. in the dihedral angle between two l.s. planes) are estimated using the full covariance matrix. The cell s.u.'s are taken into account individually in the estimation of s.u.'s in distances, angles and torsion angles; correlations between s.u.'s in cell parameters are only used when they are defined by crystal symmetry. An approximate (isotropic) treatment of cell s.u.'s is used for estimating s.u.'s involving l.s. planes.
Refinement. Refinement of *F*^2^ against ALL reflections. The weighted *R*-factor *wR* and goodness of fit *S* are based on *F*^2^, conventional *R*-factors *R* are based on *F*, with *F* set to zero for negative *F*^2^. The threshold expression of *F*^2^ > 2σ(*F*^2^) is used only for calculating *R*-factors(gt) *etc*. and is not relevant to the choice of reflections for refinement. *R*-factors based on *F*^2^ are statistically about twice as large as those based on *F*, and *R*- factors based on ALL data will be even larger.

### Fractional atomic coordinates and isotropic or equivalent isotropic displacement parameters (Å^2^)

**Table:**

	*x*	*y*	*z*	*U*_iso_*/*U*_eq_	
C1	0.4043 (2)	0.70567 (12)	0.09969 (11)	0.0437 (4)	
C2	0.2961 (2)	0.62946 (13)	0.13350 (11)	0.0488 (5)	
C3	0.2247 (2)	0.54873 (14)	0.08716 (13)	0.0563 (5)	
H3	0.153	0.4979	0.1096	0.068*	
C4	0.2590 (2)	0.54310 (14)	0.00842 (12)	0.0562 (5)	
H4	0.2107	0.4882	−0.0221	0.067*	
C5	0.3644 (2)	0.61811 (14)	−0.02606 (12)	0.0516 (5)	
C6	0.4357 (2)	0.69979 (13)	0.02022 (11)	0.0472 (5)	
H6	0.5056	0.7513	−0.0028	0.057*	
C7	0.4814 (2)	0.79400 (13)	0.15008 (11)	0.0475 (5)	
H7A	0.396	0.8424	0.1699	0.057*	
H7B	0.5543	0.8383	0.1178	0.057*	
C8	0.5769 (2)	0.74773 (14)	0.21872 (12)	0.0496 (5)	
O9	0.67141 (17)	0.66858 (11)	0.21107 (9)	0.0710 (4)	
N10	0.5600 (2)	0.80014 (13)	0.28703 (10)	0.0532 (4)	
H10	0.485 (3)	0.8525 (17)	0.2849 (12)	0.073 (7)*	
C11	0.6334 (3)	0.76130 (16)	0.35966 (12)	0.0607 (5)	
H11A	0.6477	0.8236	0.395	0.073*	
H11B	0.741	0.731	0.3484	0.073*	
C12	0.5315 (3)	0.67421 (17)	0.39979 (13)	0.0715 (6)	
H12A	0.421	0.7022	0.4064	0.086*	
H12B	0.5258	0.6094	0.3662	0.086*	
C13	0.5972 (2)	0.64031 (15)	0.47870 (12)	0.0544 (5)	
C14	0.5678 (3)	0.70159 (14)	0.54505 (13)	0.0630 (6)	
H14	0.5077	0.7668	0.5407	0.076*	
C15	0.6242 (3)	0.66973 (15)	0.61796 (13)	0.0624 (6)	
H15	0.601	0.7126	0.662	0.075*	
C16	0.7159 (2)	0.57352 (14)	0.62542 (12)	0.0514 (5)	
C17	0.7467 (3)	0.51175 (15)	0.56022 (12)	0.0623 (5)	
H17	0.8067	0.4465	0.5645	0.075*	
C18	0.6895 (3)	0.54522 (16)	0.48775 (13)	0.0671 (6)	
H18	0.7137	0.5026	0.4437	0.081*	
O19	0.7706 (2)	0.54495 (11)	0.69866 (9)	0.0700 (4)	
H19	0.794 (4)	0.475 (2)	0.7052 (16)	0.139 (12)*	
O20	0.26868 (18)	0.64349 (9)	0.21211 (8)	0.0633 (4)	
C21	0.1722 (3)	0.56269 (18)	0.25170 (14)	0.0856 (8)	
H21A	0.162	0.5826	0.3061	0.128*	
H21B	0.2242	0.4916	0.2475	0.128*	
H21C	0.0655	0.5594	0.2282	0.128*	
O22	0.38811 (19)	0.60474 (12)	−0.10495 (9)	0.0749 (5)	
C23	0.4980 (3)	0.6780 (2)	−0.14310 (14)	0.0836 (7)	
H23A	0.5043	0.6595	−0.1979	0.125*	
H23B	0.6047	0.6714	−0.1198	0.125*	
H23C	0.4595	0.7527	−0.1375	0.125*	

### Atomic displacement parameters (Å^2^)

**Table:**

	*U*^11^	*U*^22^	*U*^33^	*U*^12^	*U*^13^	*U*^23^
C1	0.0431 (10)	0.0391 (8)	0.0488 (13)	0.0005 (8)	−0.0018 (9)	0.0023 (8)
C2	0.0517 (11)	0.0435 (9)	0.0512 (13)	−0.0025 (8)	0.0037 (10)	0.0049 (9)
C3	0.0487 (11)	0.0460 (9)	0.0743 (15)	−0.0108 (9)	0.0024 (11)	0.0050 (10)
C4	0.0532 (12)	0.0503 (10)	0.0651 (15)	−0.0140 (10)	−0.0054 (11)	−0.0081 (9)
C5	0.0463 (10)	0.0565 (10)	0.0521 (14)	−0.0034 (9)	−0.0060 (10)	−0.0046 (9)
C6	0.0427 (10)	0.0465 (9)	0.0523 (13)	−0.0066 (8)	−0.0032 (9)	0.0015 (8)
C7	0.0550 (12)	0.0409 (8)	0.0465 (12)	−0.0047 (8)	0.0034 (9)	0.0004 (8)
C8	0.0484 (11)	0.0423 (8)	0.0582 (14)	−0.0069 (9)	0.0022 (10)	−0.0024 (9)
O9	0.0680 (9)	0.0593 (8)	0.0858 (11)	0.0182 (7)	−0.0068 (8)	−0.0132 (8)
N10	0.0631 (11)	0.0482 (8)	0.0483 (11)	0.0020 (9)	−0.0041 (9)	0.0003 (8)
C11	0.0649 (13)	0.0622 (11)	0.0552 (13)	−0.0086 (10)	−0.0084 (11)	0.0006 (10)
C12	0.0661 (13)	0.0735 (13)	0.0750 (16)	−0.0123 (12)	−0.0153 (12)	0.0217 (11)
C13	0.0456 (11)	0.0557 (10)	0.0618 (14)	−0.0060 (9)	−0.0070 (10)	0.0102 (10)
C14	0.0607 (13)	0.0487 (10)	0.0795 (17)	0.0101 (10)	0.0030 (12)	0.0125 (11)
C15	0.0743 (14)	0.0474 (10)	0.0656 (15)	0.0058 (10)	0.0069 (12)	−0.0013 (10)
C16	0.0557 (12)	0.0453 (9)	0.0532 (13)	−0.0073 (9)	−0.0089 (10)	0.0053 (9)
C17	0.0690 (13)	0.0551 (10)	0.0628 (14)	0.0164 (10)	−0.0085 (13)	−0.0018 (11)
C18	0.0808 (16)	0.0606 (11)	0.0600 (15)	0.0111 (12)	−0.0088 (12)	−0.0068 (11)
O19	0.0949 (11)	0.0539 (8)	0.0611 (10)	−0.0033 (8)	−0.0192 (9)	0.0029 (7)
O20	0.0770 (9)	0.0559 (7)	0.0569 (9)	−0.0153 (7)	0.0127 (8)	0.0061 (7)
C21	0.113 (2)	0.0654 (13)	0.0782 (17)	−0.0159 (14)	0.0298 (15)	0.0162 (12)
O22	0.0808 (11)	0.0891 (10)	0.0548 (10)	−0.0292 (9)	0.0034 (8)	−0.0165 (8)
C23	0.0788 (17)	0.1105 (18)	0.0614 (16)	−0.0286 (15)	0.0078 (13)	−0.0088 (13)

### Geometric parameters (Å, º)

**Table:**

C1—C6	1.378 (2)	C12—H12A	0.97
C1—C2	1.399 (2)	C12—H12B	0.97
C1—C7	1.506 (2)	C13—C14	1.371 (3)
C2—O20	1.367 (2)	C13—C18	1.382 (3)
C2—C3	1.382 (3)	C14—C15	1.378 (3)
C3—C4	1.371 (3)	C14—H14	0.93
C3—H3	0.93	C15—C16	1.387 (3)
C4—C5	1.380 (3)	C15—H15	0.93
C4—H4	0.93	C16—C17	1.360 (3)
C5—O22	1.366 (2)	C16—O19	1.368 (2)
C5—C6	1.390 (2)	C17—C18	1.379 (3)
C6—H6	0.93	C17—H17	0.93
C7—C8	1.511 (3)	C18—H18	0.93
C7—H7A	0.97	O19—H19	0.87 (3)
C7—H7B	0.97	O20—C21	1.423 (2)
C8—O9	1.235 (2)	C21—H21A	0.96
C8—N10	1.331 (2)	C21—H21B	0.96
N10—C11	1.452 (2)	C21—H21C	0.96
N10—H10	0.88 (2)	O22—C23	1.417 (2)
C11—C12	1.504 (3)	C23—H23A	0.96
C11—H11A	0.97	C23—H23B	0.96
C11—H11B	0.97	C23—H23C	0.96
C12—C13	1.503 (3)		
			
C6—C1—C2	119.14 (16)	C11—C12—H12A	108.9
C6—C1—C7	121.17 (15)	C13—C12—H12B	108.9
C2—C1—C7	119.67 (17)	C11—C12—H12B	108.9
O20—C2—C3	125.22 (16)	H12A—C12—H12B	107.7
O20—C2—C1	115.11 (15)	C14—C13—C18	116.86 (18)
C3—C2—C1	119.67 (18)	C14—C13—C12	121.78 (18)
C4—C3—C2	120.45 (17)	C18—C13—C12	121.36 (19)
C4—C3—H3	119.8	C13—C14—C15	122.16 (17)
C2—C3—H3	119.8	C13—C14—H14	118.9
C3—C4—C5	120.68 (17)	C15—C14—H14	118.9
C3—C4—H4	119.7	C14—C15—C16	119.76 (19)
C5—C4—H4	119.7	C14—C15—H15	120.1
O22—C5—C4	115.38 (16)	C16—C15—H15	120.1
O22—C5—C6	125.57 (17)	C17—C16—O19	123.00 (17)
C4—C5—C6	119.05 (18)	C17—C16—C15	118.94 (18)
C1—C6—C5	120.99 (17)	O19—C16—C15	118.06 (19)
C1—C6—H6	119.5	C16—C17—C18	120.41 (18)
C5—C6—H6	119.5	C16—C17—H17	119.8
C1—C7—C8	113.21 (13)	C18—C17—H17	119.8
C1—C7—H7A	108.9	C17—C18—C13	121.8 (2)
C8—C7—H7A	108.9	C17—C18—H18	119.1
C1—C7—H7B	108.9	C13—C18—H18	119.1
C8—C7—H7B	108.9	C16—O19—H19	115.6 (19)
H7A—C7—H7B	107.7	C2—O20—C21	117.97 (15)
O9—C8—N10	121.66 (18)	O20—C21—H21A	109.5
O9—C8—C7	121.78 (18)	O20—C21—H21B	109.5
N10—C8—C7	116.48 (16)	H21A—C21—H21B	109.5
C8—N10—C11	123.19 (17)	O20—C21—H21C	109.5
C8—N10—H10	112.2 (14)	H21A—C21—H21C	109.5
C11—N10—H10	123.7 (14)	H21B—C21—H21C	109.5
N10—C11—C12	112.56 (16)	C5—O22—C23	117.79 (15)
N10—C11—H11A	109.1	O22—C23—H23A	109.5
C12—C11—H11A	109.1	O22—C23—H23B	109.5
N10—C11—H11B	109.1	H23A—C23—H23B	109.5
C12—C11—H11B	109.1	O22—C23—H23C	109.5
H11A—C11—H11B	107.8	H23A—C23—H23C	109.5
C13—C12—C11	113.50 (18)	H23B—C23—H23C	109.5
C13—C12—H12A	108.9		

### Hydrogen-bond geometry (Å, º)

**Table:**

*D*—H···*A*	*D*—H	H···*A*	*D*···*A*	*D*—H···*A*
N10—H10···O19^i^	0.88 (2)	2.16 (2)	3.023 (2)	165.4 (19)
O19—H19···O9^ii^	0.87 (3)	1.76 (3)	2.6289 (19)	174 (3)

Symmetry codes: (i) *x*−1/2, −*y*+3/2, −*z*+1; (ii) −*x*+3/2, −*y*+1, *z*+1/2.
